# Experimental Infection of Mice and Ticks with the Human Isolate of *Anaplasma phagocytophilum* NY-18

**DOI:** 10.3390/pathogens11070820

**Published:** 2022-07-21

**Authors:** Veronika Urbanová, Eliška Kalinová, Petr Kopáček, Radek Šíma

**Affiliations:** 1 Institute of Parasitology, Biology Centre of the Czech Academy of Sciences, Branišovská 1160/31, 37005 České Budějovice, Czech Republic; veronika@paru.cas.cz (V.U.); ekalinova@post.cz (E.K.); kopajz@paru.cas.cz (P.K.); 2 Faculty of Science, University of South Bohemia, Branišovská 1645/31a, 37005 České Budějovice, Czech Republic; 3 Bioptická Laboratoř, s.r.o., Mikulášské nám. 4, 32600 Plzeň, Czech Republic

**Keywords:** *Anaplasma phagocytophilum*, tick, *Ixodes ricinus*, *Ixodes scapularis*, transmission, vector competence, animal model, human granulocytic anaplasmosis

## Abstract

*Anaplasma phagocytophilum* is the causative agent of tick-borne fever (TBF) and human granulocytic anaplasmosis (HGA) and is currently considered an emerging disease in the USA, Europe, and Asia. The increased prevalence of *A. phagocytophilum* as a human pathogen requires the detailed characterization of human isolates and the implementation of appropriate animal models. In this study, we demonstrated that the dynamics of infection with the human isolate of *A. phagocytophilum* NY-18 was variable in three different strains of mice (SCID, C3H/HeN, BALB/c). We further evaluated the ability of *Ixodes ricinus* to acquire and transmit *A. phagocytophilum* NY-18 and compared it with *Ixodes scapularis*. Larvae of both tick species effectively acquired the pathogen while feeding on infected mice. The infection rates then decreased during the development to nymphs. Interestingly, molted *I. ricinus* nymphs were unable to transmit the pathogen to naïve mice, which contrasted with *I. scapularis*. The results of our study suggest that *I. ricinus* is not a competent vector for the American human *Anaplasma* isolate. Further studies are needed to establish reliable transmission models for *I. ricinus* and European human isolate(s) of *A. phagocytophilum*.

## 1. Introduction

*Anaplasma phagocytophilum* is a tick-borne intracellular pathogen that causes diseases with varying symptoms in many animals, including humans [1]. Specifically, *A. phagocytophilum* is the causative agent of tick-borne fever (TBF), a disease with a significant economic impact on domestic ruminants, and human granulocytic anaplasmosis (HGA), an emerging zoonotic disease in the USA, Europe, and Asia [2,3]. In the vertebrate host, *A. phagocytophilum* infects neutrophils where the pathogen multiplies within a parasitophorous vacuole or morula [4].

Globally, the primary vectors of *A. phagocytophilum* are ticks belonging to the *Ixodes ricinus* complex, including *I. ricinus* in Europe, *I. persulcatus* in Asia, *I. scapularis* in eastern North America, and *I. pacificus* in western North America [5]. It is generally accepted that *A. phagocytophilum* is not transovarially transmitted. Ticks acquire the pathogen during the larval or nymphal stage when feeding on infected vertebrate hosts and maintain infection through molting to the next life stage. *A. phagocytophilum* initially infects tick midgut cells and subsequently develops in the salivary glands from where it is transmitted to susceptible hosts during the next tick feeding [6,7].

The epidemiological cycles of *A. phagocytophilum* are complex and involve a variety of vectors and mammalian hosts. Moreover, the epidemiology of *A. phagocytophilum* infection differs considerably between Europe and the USA. These differences in epidemiology are associated with significant variations in bacterial strains [8,9]. The *A. phagocytophilum* species can be divided into several genetic variants that are likely to be involved in different epidemiological cycles. Genetic analyses have revealed remarkable strain variation among *A. phagocytophilum* variants and sequence differences among isolates from ruminants, horses, dogs, and humans [10,11]. A recent study suggests that genetic clades of North American *A. phagocytophilum* differ in host specificity for rodents, humans, and deer [12].

The increased prevalence of *A. phagocytophilum* as a human pathogen requires the detailed characterization of human isolates and therefore the implementation of appropriate animal models. Laboratory mice have been used as small animal models for several tick-transmitted diseases, including Lyme borreliosis, tick-borne encephalitis, and babesiosis [13,14]. Human isolates of *A. phagocytophilum* have traditionally been studied in a sheep model. Initial studies confirmed that sheep are susceptible to infection and serve as a source for infection of *I. scapularis* ticks [15,16]. However, unlike mice, handling sheep is complicated. Moreover, it is impractical to use a representative number of sheep in experiments to account for inter-animal variability. Therefore, there is an effort to introduce transmission models involving smaller laboratory animals such as mice. Mice have been used as a model system for *A. phagocytophilum* and *I. scapularis* in North America [17]; however, experimental studies on mice and European strains transmitted by *I. ricinus* are lacking.

In this study, we compared the susceptibility of different strains of mice to infection with the human isolate of *A. phagocytophilum*. We also tested different modes of infection of mice and acquisition and transmission of the human isolate by *I. scapularis* and *I. ricinus* ticks.

## 2. Results

### 2.1. Susceptibility of Mice to Infection with A. phagocytophilum NY-18

To test the suitability of laboratory mice as an animal model for anaplasmosis transmission we examined the infectivity of the human *A. phagocytophilum* isolate NY-18 to the commonly used laboratory mice, namely C3H/HeN, BALB/c, and SCID mice, and compared the acquisition and transmission of this strain by *I. scapularis* and *I. ricinus* ticks.

**Figure 1:**
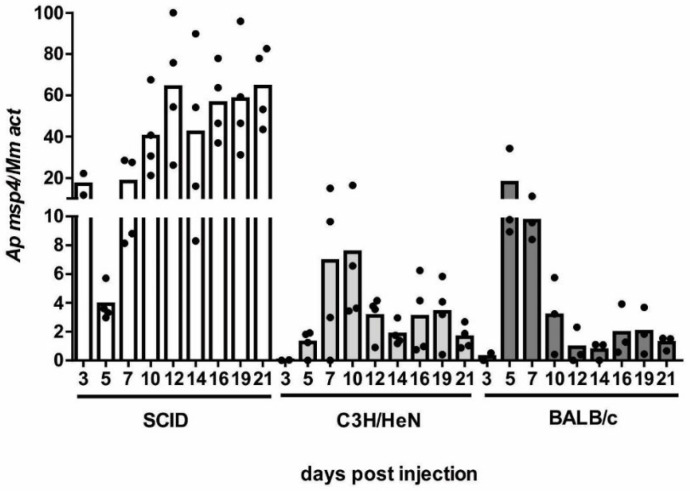
The course of *A. phagocytophilum* infection in different strains of mice. Mice were injected intraperitoneally with in vitro culture of HL-60 cells infected with *A. phagocytophilum*. The course of infection was monitored by qRT-PCR every 2–3 days, starting at day 3 post-injection (dpi). Measurements were stopped at 21 dpi. Each data point represents the relative quantification of the *A. phagocytophilum msp4* gene normalized to mouse *actin*. The columns represent the mean of the individual data points. At each time-point, 3–4 mice were analyzed.

The infection showed a different course of parasitemia in different strains of mice. The most susceptible were the SCID mice where the high infection levels were detected as early as day 3 post-injection (dpi) with in vitro culture (Figure 1). Parasitemia peaked on day 12 and then remained at a constant level until the end of the observation period. In comparison, infection in C3HeH/N mice had a slower onset. It started at 5 dpi and peaked at 7 to 10 dpi. At the remaining time points, parasitemia levels then declined to about a half. A similar course of infection was observed in BALB/c mice. The highest levels of *A. phagocytophilum* in BALB/c blood were detected at 5 and 7 dpi, and parasitemia then decreased about ten-fold (Figure 1).

### 2.2. Optimization of the Infection Process in C3H/HeN Mice

**Figure 2:**
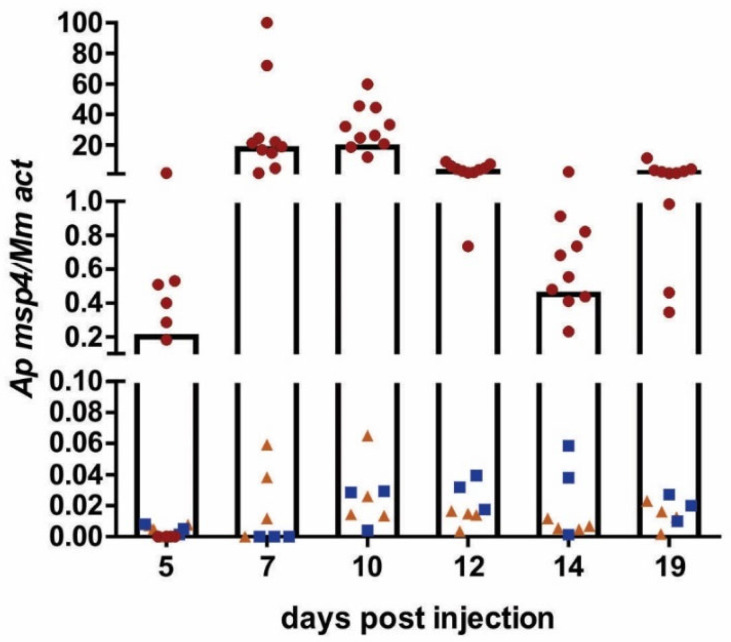
*A. phagocytophilum* infection in C3H/HeN mice. The course of infection in mice injected with (■) HL-60 cells infected with *A. phagocytophilum* from frozen stock, n = 3; (▲) in vitro culture of HL-60 cells infected with *A. phagocytophilum*, n = 4; and (●) infected blood from SCID mice, n = 10. Infection was monitored by qRT-PCR starting at 5 dpi; measurement was finished at 19 dpi. Each data point represents the relative quantification of the *A. phagocytophilum msp4* gene normalized to mouse *actin*. The columns represent the average of the individual data points for mice injected with infected blood from SCID mice. n = number of mice.

To find the best protocol for infecting mice, three different applications of *A. phagocytophilum* were tested (for details, see Section 4.4). C3H/HeN mice injected with HL-60 cells infected with *A. phagocytophilum* from frozen stocks, as well as mice injected with an in vitro culture of infected HL-60 cells, developed infection. In both groups, infection was detected at 5 dpi. Parasitemia increased slowly thereafter but remained at a negligible level throughout the follow-up period (Figure 2). 

In contrast, passage through SCID mice significantly boosted the infection level in C3H/HeN mice. Parasitemia at the peak of infection (7–12 dpi) was ~10–20-fold higher compared to mice directly injected with HL-60 culture (groups 1 and 2) (Figure 2).

### 2.3. Survival of A. phagocytophilum NY-18 in Molting Ticks

**Figure 3:**
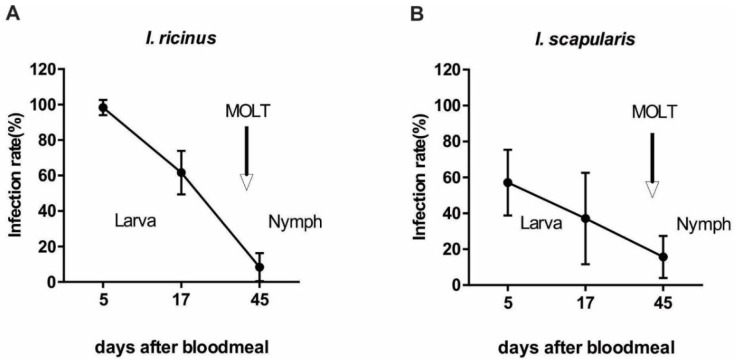
The infection rate of *A. phagocytophilum* during larval to nymphal development. (**A**) *I. ricinus*, (**B**) *I. scapularis*. Infection rates gradually decreased in both tick species during molting to nymphs. Each data point represents at least 70 individually analyzed ticks. Error bars represent mean with 95% CI.

The preparation of ticks infected with a specific pathogen is an essential step for all types of transmission studies. In the next experiment, we tested infection rate in engorged as well as molting *I. scapularis* and *I. ricinus* larvae fed on *A. phagocytophilum-*infected C3H/HeN mice. Larvae were fed on infected mice at the peaking parasitemia (8–12 dpi). Infection rates in engorged larvae were 99.3% and 58.6% for *I. ricinus* and *I. scapularis*, respectively, and then steadily decreased during molting to nymphs. The infection rate on day 17 after blood meal was 58.1% for *I. ricinus* and 37.1% for *I. scapularis*. After molting to nymphs, 8.1% of *I. ricinus* and 15.7% of *I. scapularis* ticks remained *Anaplasma*-positive (Figure 3).

### 2.4. Transmission of A. phagocytophilum NY-18 by Nymphal Ticks

**Figure 4:**
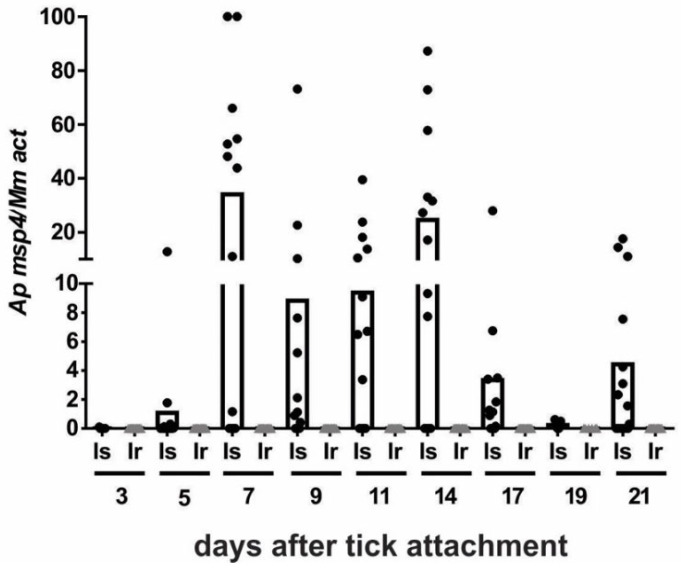
Transmission of *A. phagocytophilum* NY-18 by *I. ricinus* and *I. scapularis* nymphs. Fifteen nymphs infected with *A. phagocytophilum* NY-18 were fed on each mouse. Infection in the mouse blood was monitored every two days over a 21-day period. Each data point represents the relative quantification of the *A. phagocytophilum msp4* gene normalized to mouse *actin*. Is (●) mice (n = 13) exposed to *I. scapularis* nymphs infected with *A. phagocytophilum*, Ir (▲) mice (n = 16) exposed to *I. ricinus* nymphs infected with *A. phagocytophilum*. The columns represent the mean of the individual data points.

Infected *I. ricinus* and *I. scapularis* nymphs prepared in the previous experiment were used in a transmission experiment to test whether these ticks are capable to transmit the infection to mice. Infection in mice exposed to *I. scapularis* nymphs was first detected at 5 days post-attchment (dpa), peaked at 7 dpa and then remained relatively stable until the end of the observation period. A total of 9 of 13 mice exposed to *I. scapularis* nymphs tested positive for *A. phagocytophilum* infection. By contrast, none of the 16 mice exposed to infected *I. ricinus* nymphs developed an infection, suggesting that *I. ricinus* is not a competent vector of the American *A. phagocytophilum* isolate NY-18 (Figure 4).

## 3. Discussion

*A. phagocytophilum* is an obligate intracellular rickettsial pathogen causing human granulocytic anaplasmosis (HGA), equine and canine granulocytic anaplasmosis, and tick-borne fever (TBF) [18]. HGA is an emerging infectious disease of public health concern in North America and Europe. With demonstrated geographic range expansion of the disease vectors, the incidence of HGA disease follows in parallel [19]. 

Despite great efforts by researchers to better characterize *A. phagocytophilum* infections in Europe, several knowledge gaps remain. There are still insufficient data on the geographical distribution, host preferences, and human pathogenicity of different genetic variants of *A. phagocytophilum*. Another important aspect is to understand the differences between HGA in North America and Europe. Although there are clear differences between the ecology of North American and European strains (e.g., different vectors and hosts, and apparently different genetic variations), it is not clear whether ecology or genetic differences affect human pathogenicity or whether this may affect the ability of tick vectors to transmit the infection. The vector competence of various tick species in which *A. phagocytophilum* has been detected remains to be investigated. Given the large number of host-specific ticks, it is likely that other potential vector–host *Anaplasma* relationships will be uncovered in the future. Differences in the prevalence of *A. phagocytophilum* in ticks may be attributable to several factors, such as the susceptibility and competence of individual tick species and the susceptibility and competence of individual hosts as potential reservoirs. In particular, the availability of different reservoir hosts and the adaptive strategies of *A. phagocytophilum* appear to be critical factors in this variability. *A. phagocytophilum* is currently viewed as a single bacterial species. However, the situation appears to be more complex, as strain variation with potential host-specific tropism is apparently abundant in *A. phagocytophilum* [8]. In this study, we tested the susceptibility of different strains of mice to infection with the *A. phagocytophilum* isolate NY-18. We selected common strains of immunocompromised laboratory mice, SCID (lack of mature B and T lymphocytes), C3H/HeN (mutation in toll-like receptor 4 gene), and BALB/c (Th2-biased immune response). These strains are widely used as animal models due to their susceptibility to various infectious diseases. The NY-18 strain of *A. phagocytophilum* is a human strain originally obtained from a patient with suspected HGE residing in southeast New York State [20]. The dynamics of *A. phagocytophilum* infection in SCID, C3H/HeN, and BALB/c mice were variable after injection of the *Anaplasma*-infected HL-60 cell culture. The infection rate in C3H/HeN and BALB/c mice gradually decreased after the peak of infection. Our results are in agreement with previous studies. In the study by Hodzic et al. [21], C3H mice that were inoculated with *A. phagocytophilum* isolated from a patient with HGA developed anemia and leukopenia, but by day 24, they returned to normal values. Granulocytic morulae were present in peripheral blood and spleen smears on days 5 and 10, and there was a reduction in morulae on day 17. C3H mice were able to recover from *A. phagocytophilum* infection at 8 weeks. However, xenodiagnosis suggested that at least some C3H mice remain persistently infected for up to 55 days [21]. In a later study, C3H mice experimentally infected with *A. phagocytophilum* isolate NY-18 did not develop any clinical signs of infection. However, acute infection was associated with gross splenomegaly, microscopic inflammatory lesions in the lung and liver, hyperplastic lesions on the spleen, and clinical pathology abnormalities including neutropenia and monocytosis. Infection in mice peaked on day 10 dpi. At this time point, infection was detected in 3/3 mice. Thereafter, the infection rate decreased, with infection detected in 0/3 and 1/3 of mice on days 14 and 20 dpi, respectively [17]. 

Unlike immunocompetent mice, which eventually clear the infection, mice with severe combined immunodeficiency (SCID) remain permanently infected [21,22]. *A. phagocytophilum*-infected SCID mice are frequently used as a source of infectious material in transmission studies. Different modes of infection have been shown to have a significant effect on the development of infection in mice [21]. Culturing *A. phagocytophilum* in HL-60 cells leads to a rapid reduction in pathogenicity and infectivity of the bacteria in mice. Blood passage through SCID mice is the standard procedure for restoring the infectivity of cultured *Anaplasma* [23]. In our experiments, parasitemia was much higher in mice injected with blood from SCID mice infected with *A. phagocytophilum* compared with mice injected with in vitro or thawed HL-60 cell culture infected with *A. phagocytophilum*.

To this end, most studies in Europe have focused on assessing the distribution and estimating the prevalence of *A. phagocytophilum* in *I. ricinus* ticks. In contrast to the USA, only a few papers have been published on the transmission of *A. phagocytophilum* strains by *I. ricinus* ticks. In a study by Fourie et al. [24], *I. ricinus* ticks experimentally infected with a canine strain of *A. phagocytophilum* transmitted infection within a few hours after attachment, but the establishment of infection in dogs was dependent on the minimum inoculation dose, which was only observed if the ticks were attached for more than 48 h [24]. In another study, Almazán et al. [25] established a transmission model involving sheep, *I. ricinus* ticks, and an ovine strain of *A. phagocytophilum* (European Norwegian variant 2—NV2Os). Sheep inoculated with IDE8 tick cells infected with NV2Os strain developed *A. phagocytophilum* infection. It was manifested by fever at 4 dpi, the observation of morulae in granulocytes at 6 dpi, and the presence of *A. phagocytophilum* in various tissues at 14-15 dpi. Importantly, *I. ricinus* ticks fed on infected sheep were able to maintain *Anaplasma* infection through molting; 70% of nymphs and 90 % of adults fed on sheep during the acute phase of infection were positive for *A. phagocytophilum*. Infected nymphs were then able to transmit the bacteria to naïve sheep. In contrast, only 2.7% of nymphs engorged as larvae during the persistent phase were *Anaplasma*-positive, suggesting the importance of proper timing of infestation [25].

These experimental models will undoubtedly facilitate future research on European *Anaplasma* strains and *I. ricinus*. Nevertheless, no studies have been published to date on the human strain of *Anaplasma* and *I. ricinus* as a vector. To our best knowledge, the only study testing the infectivity of human *A.* *phagocytophilum* NY-18 for *I. ricinus* ticks was presented by Kazimirova et al., at the “IV. Labudove dni” conference in 2015 but has not yet been published [26]. Therefore, we evaluated the ability of *I. ricinus* to acquire and transmit human *A. phagocytophilum* isolate NY-18 and compared it with *I. scapularis*. *I. ricinus* larvae effectively acquired the pathogen while feeding on infected mice. A total of 99.3% of engorged *I. ricinus* larvae tested positive for *A. phagocytophilum*. The infection rate then decreased during the development to nymphs. After molting, only 8.1% of nymphs were positive. A similar decrease in infection rate was observed also during transstadial development of *I. scapularis*. A total of 58.6% of *I. scapularis* larvae acquired the infection and after molting, while 15.7% of *I. scapularis* nymphs remained *Anaplasma*-positive. Similar acquisition percentages in field populations of *I. ricinus* have been reported in a study by Ogden et al. [27]. Sheep naturally exposed to tick-infested pasture for a minimum of 4 weeks, resulted in 59 and 72% of PCR-positive engorged larvae and nymphs, respectively. In this study, the authors reported that after molting, engorged larvae led to 18.5% of infected nymphs [27]. 

Interestingly, molted *I. ricinus* nymphs were unable to transmit the pathogen to naïve mice, which contrasted with *I. scapularis*. Nine of thirteen (69%) mice exposed to *I. scapularis* nymphs tested positive for *A. phagocytophilum* infection. The results of our study suggest that *I. ricinus* is not a competent vector for the American human *Anaplasma* isolate. Our observation that the specific tick species can imbibe *Anaplasma*-containing blood from an experimentally infected animal but is unable to transmit the infection to the next host is consistent with a report documenting the identification of *A. phagocytophilum* in *Haemaphysalis longicornis* ticks that fed on experimentally infected goats [28]. However, the observation that *H. longicornis* is not competent to transmit *A. phagocytophilum* is not surprising because only ticks of the genus *Ixodes*, specifically ticks belonging to the *I. ricinus* complex, have been confirmed as primary vectors of *A. phagocytophilum*. 

## 4. Materials and Methods

### 4.1. Ticks and Mice

The *I. ricinus* ticks used in this study were obtained from the breeding facility of the Institute of Parasitology, Biology Centre, Czech Academy of Sciences. Adult and nymphal *I. scapularis* ticks were kindly provided by Dr. Michael L. Levin (CDC, Atlanta, GA, USA) and obtained through the NIH Biodefense and Emerging Infections Research Resources Repository, NIAID, NIH (BEI Resources, Cat. Nos. NR-42510 and NR-44116, respectively). Both tick species were kept in humid chambers with 95 % humidity, 24 °C temperature, and day/night period set to 15/9 h.

Inbred, pathogen-free immunodeficient SCID mice were obtained from the Animal Facility of the Institute of Parasitology, Biology Centre, Czech Academy of Sciences. The inbred, pathogen-free BALB/c and C3H/HeN mice (The Jackson Laboratory, Bar Harbor, ME, USA) were purchased from Anlab (Prague, Czech Republic). Mice were used for experimental infections of *I. scapularis* or *I. ricinus* larvae and for transmission experiments.

### 4.2. Anaplasma phagocytophilum In Vitro Culture

*A. phagocytophilum* isolate NY-18 [17] (kindly provided by Prof. José de la Fuente, University of Castilla-La Mancha, Ciudad Real, Spain) was propagated in HL-60 promyelocytic cell line (ATCC, CCL-240) in RPMI 1640 culture medium (GIBCO) containing 2% MEM amino acid solution (GIBCO) and 10% heat-inactivated (56 °C, 30 min) fetal calf serum (FCS, Lonza) at 37 °C with 5% CO_2_ [29]. The number of infected cells was monitored under an Olympus BX53 microscope. The cell smear was air-dried and stained with RAL DIFF-QUIK (Siemens Healthineers, Erlangen, Germany) according to the manufacturer’s instructions. Cultures were injected into mice when ~ 50% of cells were infected.

### 4.3. Preparation of Infected Mice and Ticks

A volume of 10 mL of *A. phagocytophilum* in vitro culture was centrifuged for 5 min at 300× *g*. Most of the supernatant was aspirated, and the pellet was resuspended in approximately 1.5 mL of the remaining supernatant. Each mouse was then injected intraperitoneally (i.p.) with 200 µL of concentrated culture. 

The frozen culture of *A. phagocytophilum* infected HL-60 cells was thawed at 37 °C, centrifuged for 5 min at 300× *g*, and washed 2 times with a fresh culture medium. Mice were injected i.p. with 200 µL of the culture.

Mouse-to-mouse blood transfer was performed as follows. *Anaplasma*-positive SCID mice were anesthetized with a mixture of 5% Narkamon (Spofa), and 2% Rometar (Spofa) in 1× PBS (Phosphate Buffered Saline, pH 7.3). Blood was collected into a citrate–phosphate–dextrose solution (Sigma Aldrich, St. Louis, MO, USA) (ratio 14:1) to prevent coagulation. Six-week-old female C3H/HeN mice were injected i.p. with 200 µL of blood. 

To prepare *A. phagocytophilum* infected nymphs, clean *I. ricinus* or *I. scapularis* larvae were fed on C3H/HeN mice infected with *A. phagocytophilum* and allowed to molt into nymphs. The infection rate of the nymphs was examined by PCR (see below). Fifteen potentially *A. phagocytophilum* infected nymphs (*I. ricinus* or *I. scapularis*) were then fed on naïve C3H/HeN mice to test the ability of the ticks to transmit infection. Infection in the mouse blood was monitored every two days starting at 3 dpa, and observation was terminated at 21 dpa.

### 4.4. Optimization of the Infection Process in C3H/HeN Mice

Three different applications of *A. phagocytophilum* to C3H/HeN mice were tested: (1) C3H/HeN mice were injected with HL-60 cells infected with *A. phagocytophilum* directly from frozen stocks. The culture was thawed and immediately injected into the mice; (2) C3H/HeN mice were injected with an in vitro culture of HL-60 cells infected with *A. phagocytophilum*; (3) in vitro culture of HL-60 cells infected with *A. phagocytophilum* was first injected into SCID mice. Then, at 14 dpi, blood from infected SCID mice were passaged to the naïve C3H/HeN mice.

### 4.5. DNA Isolation

DNA from mouse blood collected from cut tails or tick homogenates was isolated using DNeasy Blood & Tissue Kit (Qiagen, Hilden, Germany) according to the manufacturer’s protocol. Briefly, ticks were individually placed in a 2 mL Screw Cap Tube (BIOplastics, Landgraaf, Netherlands) with 3 pieces of Triple-Pure Zirconium Beads (Benchmark Scientific). Ticks were homogenized in MagNa Lyser (Roche, Basel, Switzerland) for 90 s, 300× *g*. Then, 180 µL of ATL tissue lysis buffer with 20 μL of proteinase K was added, and samples were vortexed and incubated at 56 °C overnight.

### 4.6. PCR and Real-Time qPCR

Infection of *A.* *phagocytophilum* in ticks and mouse blood was verified by PCR. The reaction mixture consisted of 12.5 µL Fast Start Mastermix (Roche), 10 pmol of AP-F and AP-R primers (Table 1), 4 µL of DNA, and PCR water up to 25 µL. PCR products were visualized on a 2% agarose gel (Sigma Aldrich) stained with ethidium bromide.

The relative loads of *A.* *phagocytophilum* in mouse tissues were analyzed by quantitative real-time PCR (qRT-PCR) amplifying the *msp4* gene. The reaction mixture contained 12.5 µL of the FastStart Universal SYBR Green Master (Roche), 10 pmol of primers AP-F and AP-R (Table 1), 4 µL of DNA, and PCR water up to 25 µL. The amplification program consisted of denaturation at 95 °C for 10 min, followed by 50 cycles of denaturation at 95 °C for 20 s, annealing at 60 °C for 20 s, and elongation at 72 °C for 30 s. Each assay was performed in technical triplicates.

Quantification of mouse and tick *actin* was performed using the primers Mm-F and Mm-R, Ir-F, and Ir-R (Table 1). Reaction and amplification conditions were the same as described above.

Relative levels of the *A. phagocytophilum* target gene (*msp4*) was normalized to mouse and tick actin using the mathematical model of Pfaffl [30].

## 5. Conclusions

In this study we demonstrated that common laboratory mice are suitable model hosts for the human isolate of *A. phagocytophilum*. However, the dynamics of infection varies between different strains of mice. We further demonstrated that both tick species, *I. ricinus* and *I. scapularis*, effectively acquire *A. phagocytophilum* when fed on infected mice. Interestingly, *I. ricinus* nymphs are not able to transmit the pathogen to naïve mice, which is in contrast with *I. scapularis*. Our results suggest a highly specific adaptation of *A. phagocytophilum* to its tick vector. Further studies are needed to establish reliable transmission models for *I. ricinus* and European human isolate(s) of *A. phagocytophilum*.

## Figures and Tables

**Table 1:** Primers used in this study.

Organism	Gene	Primer Name	Annealing Temp (°C)	Sequence 5′→3′
*Mus musculus*	*actin*	Mm-F	60	AGAGGGAAATCGTGCGTGAC
		Mm-R	60	CAATAGTGATGACCTGGCCGT
		Mm-P	60	CACTGCCGCATCCTCTTCCTCCC
*Ixodes ricinus*	*actin*	Ir-F	60	GAGGCATGAGGGTGTGTTTT
		Ir-R	60	GACCTGCACGAAAATGATTG
*Anaplasma phagocytophilum*	*msp4*	Ap-F	60	TGACAGGGGAGGATCTTACG
		Ap-R	60	TCTAGCTCCGCCAATAGCAT

## Data Availability

Not applicable.
